# The Use of Mesenchymal Stem/Stromal Cell-Derived Extracellular Vesicles in the Treatment of Osteoarthritis: Insights from Preclinical Studies

**DOI:** 10.3390/bioengineering11100961

**Published:** 2024-09-26

**Authors:** Mitch Jones, Elena Jones, Dimitrios Kouroupis

**Affiliations:** 1 Department of Chemistry, Loughborough University, Loughborough LE11 3TU, UK; mitch.jones0508@gmail.com; 2 Leeds Institute of Rheumatic and Musculoskeletal Disease, University of Leeds, Leeds LS2 9JT, UK; 3 Department of Orthopedics, UHealth Sports Medicine Institute, Miller School of Medicine, University of Miami, Miami, FL 33146, USA; 4 Diabetes Research Institute & Cell Transplant Center, Miller School of Medicine, University of Miami, Miami, FL 33136, USA

**Keywords:** mesenchymal stem/stromal cell-derived extracellular vesicles, osteoarthritis, cartilage regeneration, inflammation attenuation

## Abstract

Osteoarthritis (OA) is a prominent cause of disability, and has severe social and economic ramifications across the globe. The main driver of OA’s pervasiveness is the fact that no current medical interventions exist to reverse or even attenuate the degeneration of cartilage within the articular joint. Crucial for cell-to-cell communication, extracellular vesicles (EVs) contribute to OA progression through the delivery of bioactive molecules in the inflammatory microenvironment. By repurposing this acellular means of signal transmission, therapeutic drugs may be administered to degenerated cartilage tissue in the hopes of encouraging regeneration. Positive outcomes are apparent in in vivo studies on this subject; however, for this therapy to prove itself in the clinical world, efforts towards standardizing the characterization, application, biological contents, and dosage are essential.

Osteoarthritis (OA) affects approximately 500 million people worldwide and is a common cause of disability and societal economic burden [1]. It is a chronic degenerative disease of the joints characterized by an irrecoverable loss of articular cartilage, low-grade synovial inflammation, and bone hypertrophy, primarily in the knee, hip, hands, and spine [2]. OA has a complex etiology depending on the initial triggers and genetic and environmental risk factors. Multiple OA phenotype classifications have been proposed to account for disease heterogeneity. However, regardless of the phenotype, patients have similar clinical endpoints (pain, stiffness, loss of function), and a common activation of low-grade joint inflammatory/immune cascades [3,4,5]. Conventional drug therapies are capable of controlling pain; however, they have not been shown to have a sustained effect on the long-term mitigation of OA symptoms or disease progression and can have deleterious effects when prescribed over long durations [6]. With this in mind, research and clinical efforts have been directed towards the development of OA therapies that do not just alleviate symptoms but target the joint proinflammatory area early following the onset of degeneration.

Newer, regeneration-oriented interventions, including orthobiologic therapy with mesenchymal stem/stromal cells (MSCs) and their derivatives, such as extracellular vesicles (EVs), require standardization, stricter regulation, and further evidence of clinical efficacy [7,8,9,10]. Deemed “injury drugstores”, MSCs have been shown to have anti-inflammatory, antifibrotic, anabolic, and analgesic therapeutic activity, all of which could prove beneficial to patients with OA [11]. While numerous in vitro studies have shown MSC-EVs’ beneficial effects on cultured chondrocytes and immune lineage cells, preclinical evidence remains at an early, though crucial, stage for identifying the key steps towards clinical translation. In this commentary, we highlight the experimental techniques that have already become standard, such as the cargo and function characterization of MSC-derived EVs, as well as areas in which experimental approaches remain highly variable. These include animal models, EV tissue sources, doses, delivery methods, and treatment schedules. The benefits of delivering certain exogenous cargos, such as microRNAs, over native EVs, are yet to be explored. The utilization of animal models, representing different etiologies of human OA, would help to rationally design clinical trials targeting different groups of OA sufferers.

Extracellular vesicles, crucial for physiological cell-to-cell communication, are seen as a new frontier in OA therapeutics, owing to their acellular nature, small sizes, and ability to transport bioactive molecules. While MSCs do secrete cytokines and chemokines directly, they also exhibit their paracrine effects via EVs. According to the International Society of Extracellular Vesicles (ISEV) guidelines, EVs are defined as “particles that are released from cells, are delimited by a lipid bilayer, and cannot replicate on their own” [12]. Specifically, EVs serve as vehicles for cellular export products, including lipids, proteins, and RNAs (mRNAs and miRNAs), and can modulate the function of other cells at proximal or distal sites [13]. Specifically deployed ‘therapeutic’ MSC-EVs, derived from healthy tissues or purposefully designed to carry specific cargos, can deliver restorative compounds to the site of damage and, in relation to OA, may aid in joint regeneration. The proposed mechanisms of action of MSC-EVs on an OA environment include the following: the regulation of chondrocytes’ senescence and apoptosis, inducing favorable changes in their bioenergetic metabolism, the enhancement of their extracellular matrix production, and regulating the immune responses in the synovium [14,15,16].

EV therapy, although only recently gaining clinical interest, has several benefits over current cell-based regenerative approaches, such as platelet-rich plasma (PRP) or MSC therapies (Figure 1). Compared to an autologous PRP [17], EVs would be produced under highly controlled good manufacturing practice (GMP) conditions [18], where quality control (QC) standards already exist. The potential limitations of MSC therapy, such as the risk of immuno-rejection, tumorigenicity, and short in situ lifespans are overcome by utilizing acellular particles instead of living cells [19]. Furthermore, the ability to produce EVs from immortalized cell lines instead of primary cells also reduces batch-to-batch variability, aids in QC assessments, and reduces overall costs [20].

Thus far, numerous in vitro studies have succeeded in demonstrating the effectiveness of MSC-EVs in reducing OA-like chondrocyte apoptosis, pyroptosis, and the release of proinflammatory and catabolic molecules, as well as in increasing their viability, migration, and anabolic abilities [21,22,23,24,25]. Additionally, recent studies have shown the effectiveness of MSC-EVs in attenuating the immune lineage cells’ proinflammatory activation and pain signaling [26,27,28,29]. While the source and lineage of the host MSCs remain variable, the generation and characterization of the produced EVs normally followed the most recent ISEV guidelines [12], and the growing consensus revealed positive outcomes in the majority of in vitro studies [8,16,30,31]. Comprehensive studies of MSC-EVs cargos have identified the most promising candidate molecules for incorporating into ‘engineered EVs’, as discussed previously [16,26,32,33,34]. However, even in advanced three-dimensional in vitro culture models, complex biomechanical and pathophysiological joint environments cannot be fully reproduced [35], necessitating preclinical evaluations in animal models.

Reviews and meta-analysis studies have described the efficacy of intra-articularly (IA) injected MSC-EVs in a variety of OA animal models [15,16,35]. When it comes to the source of parental cells and animal species, the previously used approaches remain variable (Table 1). Following successful tests of EVs from the host animals’ MSCs (commonly BM-MSC-derived [36,37]), more recent studies have focused on human MSC-EVs, predominantly from the BM [23,33,38,39,40,41,42,43,44] and adipose tissues [25,28,29,45,46], but also from the synovium [47,48,49,50,51,52,53,54] or the umbilical cord [55,56]. According to these studies, the following specific cargos have been investigated and proven effective: miR-92a-3p, miR-136-5p, miR-3960, miR-125a-5p, miR-361-5p, LncRNA NEAT1, and lncRNA MEG-3 for BM-derived MSC-EVs; miR-100-5p for adipose tissue-derived MSC-EVs; miR-26a-5p, miR-155-5p, circRNA3503, miR-140-5p, and miR-31 for synovium-derived MSC-EVs; and LncRNA H19, miR-1208 and circHIPK3 for UC-MSC-EVs (Table 1). However, direct comparisons in the same study of the effectiveness of EVs or their cargos from different MSC tissue sources, for example, BM and adipose tissue, remain infrequent [57]. Although much attention has been given to nucleic acid-based cargos, few studies have investigated specific protein cargos, such as CD10 [28] and CD90 [47], and proven the effectiveness regarding the enhanced repair of damaged cartilage and their anti-inflammatory ability.

Small animals (mice and rats) represent the most commonly utilized species model, followed by rabbits [43,47] and occasionally, minipigs [62]. The doses of delivered MSC-EVs were commonly calculated as particle numbers or, less frequently, by weight, with some studies additionally reporting the numbers of parental MSCs [28,29,38]. Treatment schedules and follow-up periods differed considerably, depending on the animal model and the study’s unique protocol (Table 1). Some studies employed single IA injections; however, the majority of studies favored multiple injections at daily or weekly intervals depending on the animal model and how OA changes had been induced (enzymatically, chemically, or surgically). Control groups commonly comprised ‘sham’ (normally PBS, the same volume as EVs) and ‘OA’ (OA-induced but not treated) groups, while some studies additionally included healthy joints. Parental MSCs as a comparator group for the assessment of relative efficacy were used less frequently [38,45]. The most common follow-up periods were 4–6 weeks (for mice) and 4–8 weeks (for rats), although shorter periods were considered for post-traumatic OA models, such as oleanolic acid- or ACL- rupture-induced models (both in mice) [39,41].

As mentioned, IA injections were the most common method, but not the only method, of EV delivery. EV-loaded hydrogels are becoming an increasingly popular means of EV delivery, because while injectable, they can be combined with 3D bioprinting [63]. For example, a study conducted by Chen et al. used an infused hydrogel scaffold to restore mitochondrial function in dysfunctional chondrocytes, attenuate chondrocyte degeneration, and rescue degenerated cartilage in an osteochondral defect model in mice [64]. The implementation of hydrogels has the benefits of delivering MSC-EVs without rapid cargo clearance and accidental cartilage disruption, as well as targeted degradation in response to OA stimuli (such as pH and temperature) that ensures precise delivery to the specific site of injury or inflammation [64,65]. The targeting ability of EVs can also be enhanced by their surface modification. This can be achieved by the genetic modification of parental MSCs, enabling EVs to express proteins and other molecules with high affinity to surface receptors on the target cells [26,66]. The systemic delivery of EVs can also be employed to target deep layer chondrocytes and subchondral bone-resident cells, such as osteocytes and osteoclasts, that are not easily accessible when utilizing IA injections [67,68].

Outcome assessments presented fairly good uniformity and commonly measured structural improvements when utilizing the Osteoarthritis Research Society International (OARSI) or International Cartilage Repair Society (ICRS) scoring systems (Table 1). Histology and immunohistochemistry were normally used to demonstrate the increase in cartilage catabolic markers, such as collagen II and aggrecan, and reductions in cartilage catabolic enzymes, such as collagenases and aggrecanases. Similarly, because it has been well described that during the progression of OA, the synovium and infrapatellar fat pad serve as a source of pain-transmitting, immune, and inflammation-modulating neuropeptides [26,69,70], many studies have focused on the effects of EVs on inflammation and pain reversal. Specifically, IA MSC-EV delivery results in a regenerative immune phenotype characterized by a higher infiltration of M2 anti-inflammatory over M1 proinflammatory macrophages, with a concomitant reduction in IL-1β and TNF-α proinflammatory and an increase in IL-10 anti-inflammatory synovial cytokines [29,47,48,50,54,56,59]. Pain or gait analyses and measurements of systemic changes in serum biomarkers remain less frequent (Table 1). When tested, pain behavior and joint motion tended to exhibit recovery, suggesting the discernible health benefits of this treatment [45,65].

Furthermore, as stated, there are major regulatory challenges when using primary MSCs as EV-producing cells [71]. To overcome these hurdles and reduce batch-to-batch heterogeneity, immortalized primary MSCs or hES-derived MSC lines represent an attractive alternative [30,72]. Larger animal models need to be increasingly utilized and have already begun to be employed to test MSC-EVs therapies for OA. For example, in a recent minipig model study, the combination of MSC-EVs and hyaluronic acid was administered intra-articularly at weekly intervals and evidenced the promotion of functional osteochondral repair using magnetic resonance imaging and microcomputed tomography, as well as using histological and biomechanical tests [62]. The design of this study offered a clinically translatable protocol for a future clinical trial. Horses may represent another suitable large test model, as they experience gravitational impacts on joints, similar to people, and are amenable to arthroscopic intervention, diagnostic imaging, and repeated sample collection [73]. With the aim of providing an effective treatment as early as possible, and given the scarcity of disease management options for physically active people or people with high-BMI OA, utilizing mild and metabolic OA animal models would be another productive way forward [62].

There are currently five ongoing clinical trials in the MSC-EV field of OA regeneration, all targeting early-to-moderate knee OA and involving IA injections (Table 2). Being in the early phases of clinical research, the trials are primarily evaluating the potential of adverse events arising from the treatment. However, two trials have progressed to dose optimization and aim to compare clinical improvements after 1 year. Due to MSC-EV treatment being in its infancy, clinical trials have yet to publish their results.

Overall, preclinical studies show that MSC-EVs can regulate and prevent symptoms in OA, notably by restoring a healthy chondrocyte phenotype and by attenuating inflammation and pain signaling. These models are very useful for dose optimization and the development of effective material carriers and delivery methods. Future work should aim to standardize animal models, EV doses, and delivery schedules in order to ensure consistency between the studies and the selection of the most efficacious EV preparations. Three-dimensional joint models should continue to be developed, as well as novel technologies for the incorporation of further therapeutic and targeting agents, such as miRNAs and proteins, into the engineered EVs. Until recently, clinical implementation has seemed distant due to the challenges related to EVs tropism, limited native therapeutic efficacy, administration routes, bioavailability, and long-term therapeutic efficacy. At present, advanced protocols to engineer MSC-EVs and their combination with novel biomaterials have shown the significant therapeutic benefits of MSC-EVs in OA animal models. The first in-human clinical trials have been initiated to test the safety and efficacy of unmodified EVs. Therefore, MSC-EVs can be considered a promising nano-immunoregulatory and anabolic modality for musculoskeletal disease treatments and especially for OA.

## Figures and Tables

**Figure 1:**
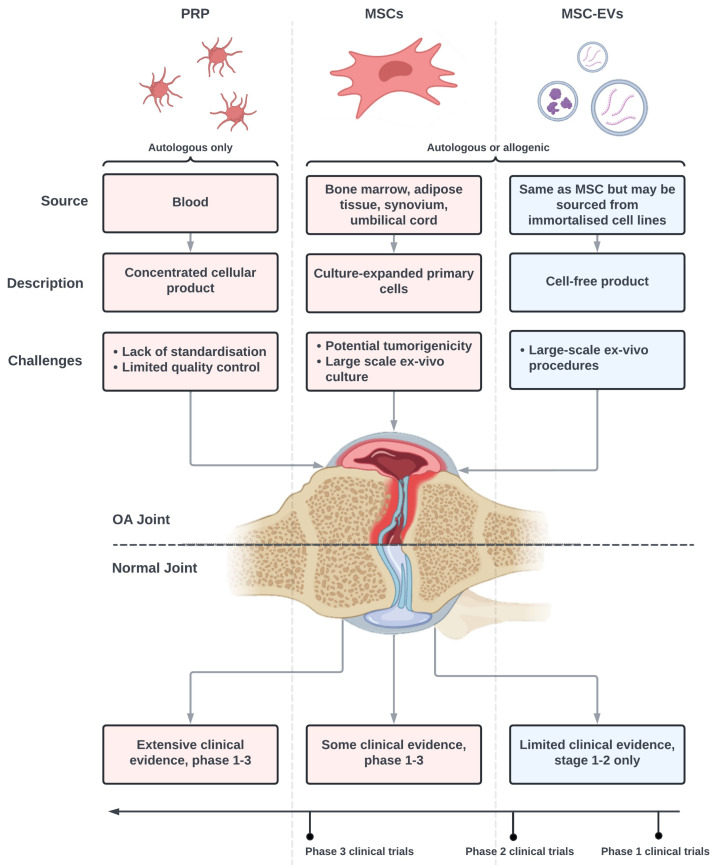
Potential advantages for MSC-EV therapy (blue) compared to platelet-rich plasma (PRP) and MSC therapies (red). PRP is a constituent of a patient’s blood (IA injected) that possesses large concentrations of platelets and regenerative factors. The source, basic description, and challenges for each therapy are given, as well as their derivation. Two of the main challenges involved in the PRP approach are circumvented by MSC-EV treatment, due to EVs being primarily donor-independent and grown and produced under good manufacturing practice (GMP) conditions. The potential tumorigenicity of MSC therapy is subverted using acellular particles instead of living cells. From this, the ability to produce EVs from immortalized cell lines instead of primary cells also reduces the batch-to-batch variability of the product. Although PRP and MSC therapy have received a greater deal of research in clinical trials, MSC-EVs have begun to undergo stage 1–2 clinical trials in OA therapeutics. Image taken from Biorender.

**Table 1:** Summary of human MSC-EV therapeutics in OA animal models.

Source	Cargo	OA Model	EVs Dose	Control Group	Follow Up Period	Outcome Measures	Key Results	Ref.
hAD-MSC	Various miRNAs	- MIA induced in rats - BMM in mice	- In rat subacute OA group: 30 μL injection once per week for 21 days of hASC-EVs (1 × 10^8^ particles) or PBS and hyaluronic acid - In rat chronic OA group: 30 μL injection twice per week for 40 days of hASC-EVs (1 × 10^8^ particles) or PBS and hyaluronic acid - 6 μL injection once per week of EVs (1 × 10^8^ particles) or PBS	PBS	4 and 8 weeks	Histology (H), immunohistochemistry (IH), OARSI scores	EVs significantly attenuated OA progression and protected cartilage from degeneration in both the monosodium iodoacetate (MIA) rat and the surgical destabilization of the medial meniscus (DMM) mouse models	[46]
hAFSC	Not defined	MIA induced in rats	100 μg, repeated after 10 days	OA, AFSC (5 × 10^5^ cells)	3 weeks	H/IH, OARSI scores, pain assessment	Enhanced pain tolerance, lower OARSI scores comparable to AFSC-treated defects	[45]
hBM-MSC	miR-92a-3p	Collagenase induced in mice	15 μL (500 μg/mL), once a week	Healthy, OA, MSC-miR-92a-3p-transfected	3 weeks	H/IH, WB	Reduced cartilage matrix loss, improved col2a1 and aggrecan expression (further improved in MSC-miR-92a-3p group	[33]
hBM-MSC	Not defined	Groove surgery in rats on high-fat diet	7.77 × 10^7^ particles (from 2 × 10^6^ MSCs), five doses with 5-day intervals	OA, PBS, 2 × 10^6^ MSCs	11weeks	µCT scans at week 0, 12 and 24, pain behavior, H/IH, OARSI scores	Unchanged OARSI scores, pain behavior and MMP13 staining in cartilage (compared to MSC group where these were unexpectedly aggravated)	[38]
hBM-MSC	miR-136-5p	Post-traumatic oleanolic acid (OA) in mice	100 μL single injection of 10^11^ particles/mL of EVs or miR-136-5p EVs	Healthy, OA	1 h	Histology (H), immunohistochemistry (IH)	miR-136-5p was found to reduce the degeneration of cartilage extracellular matrix	[39]
hBM-MSC	miR-3960	DMM in mice	10 μL MSC-EVs or sterile normal saline were injected into the articular capsule for 3 weeks (once a week)	Sham, OA, MSCs-EVs-agomir-NC	3 weeks	Histology (H), pain assessment	miR-3960 shuttled by MSC- EVs protected against apoptosis and ECM degradation in chondrocytes	[40]
hBM-MSC	miR-125a-5p	ACL rupture in mice	100 μL single injection of 10^11^ particles/mL	Healthy, OA	1 h	WB, qPCR	Alleviation in chondrocyte extracellular matrix degradation	[41]
hBM-MSC	miR-361-5p	ACL rupture in rats	250 ng/5 µL EVs-miR-NC or EVs-miR-361-5p postoperatively for 7 days	Sham, OA	8 weeks	Histology (H), WB, qPCR	miR-361-5p alleviates cartilage damage	[23]
hBM-MSC	LncRNA NEAT1	DMM in mice	10 μg EVs or an equivalent amount of PBS, twice a week for 1 month	Sham, OA, DMM + Lv − NC − BMSCs − EVs group, DMM + Lv − NEAT1 – BMSCs − EVs group, DMM + Lv − NEAT1 − BMSCs − EVs + sh − Sesn2 group	7 weeks	Histology (H), immunohistochemistry (IH), OARSI scores	LncRNA NEAT1 induced the proliferation and autophagy of chondrocytes but inhibited their apoptosis	[42]
hBM-MSC	Not defined	Osteochondral defect in rabbits	300 μL injection once a week for 4 weeks of 1 × 10^10^ particles/mL (low dosage) or 5 × 10^10^ particles/mL (high dosage) or PBS	Healthy, OA	5 weeks	Histology (H), ICRS scores	Facilitates cartilage regeneration and enhances viability of chondrocytes	[43]
hBM-MSC	lncRNA MEG-3	DMM in rats	100 µL injection per week EVs solution (100 µg) or MSCs (10^6^ cell)	Sham, OA	8 weeks	Histology (H), immunohistochemistry (IH), OARSI scores, micro-CT	- MSC and MSC-EVs alleviated cartilage destruction and subchondral bone remodeling - lncRNA MEG-3 also reduced the senescence and apoptosis of chondrocytes	[44]
hBM-MSC and hAD-MSC	Not defined	Ciproflaxin induced in mice	25 mL at 100 μg/μL, once a week	Healthy, OA, PBS	3 weeks	H/IH, OARSI scores, real-time PCR	Lower OARSI scores, upregulated COLII protein and Sox9, COL2 and Aggrecan genes in cartilage, particularly in BM-MSC group	[57]
hESC-MSC	Not defined	DMM in mice	5 μL every 3 days for 4 weeks	OA, PBS	4 weeks	Histology (H), immunohistochemistry (IH), OARSI scores	Lower OARSI scores, stronger Col II and weaker ADAMTS5 staining of cartilage	[58]
hESC-MSC	Not defined	Osteochondral defect in rats	100 μL injection once a week of 100 μg EVs or PBS	OA, PBS	12 weeks	Histology (H), immunohistochemistry (IH), multiplex cytokine array	EV-treated defects displayed a regenerative immune phenotype characterized by a higher infiltration of CD163+ regenerative M2 macrophages over CD86+ M1 macrophages, with a concomitant reduction in proinflammatory synovial cytokines IL-1β and TNF-α	[59]
hESC-MSC	Not defined	Osteochondral defect in rats	100 μL injection once a week of 100 μg EVs or PBS	OA, PBS	12 weeks	Histology (H), ICRS scores	- Enhanced gross appearance and improved histological scores - EV-treated defects displayed complete restoration of cartilage and subchondral bone with characteristic features including a hyaline cartilage with good surface regularity, complete bonding to adjacent cartilage, and extracellular matrix deposition	[60]
hIFP-MSC	miR-100-5p	DMM in mice	10 μL (10^10^ particles/mL) twice a week	OA, PBS	4 or 6 weeks	H/IH, OARSI scores, gait analysis	Lower OARSI scores, stronger Col II and weaker ADAMTS5 and MMP13 staining of cartilage, partial improvement of the gait patterns	[25]
hIFP-MSC	Various miRNAs	MIA induced in rats	50 μL single injection of EVs derived from 5 × 10^5^ and 5 × 10^6^ IFP-MSC	Healthy, OA	4 days	Histology (H), immunohistochemistry (IH)	MSC-EV therapeutic treatment resulted in robust macrophage polarization towards an anti-inflammatory therapeutic M2 phenotype within the synovium/IFP tissues	[29]
hIFP-MSC	Various miRNAs and CD10 protein	MIA induced in rats	50 μL single injection of EVs derived from 1 × 10^6^ IFP-MSC	Healthy, OA	4 days	Histology (H), immunohistochemistry (IH)	CD10High EV treatment resulted in robust chondroprotective effects by retaining articular cartilage structure/composition and PRG4 (lubricin)-expressing cartilage cells	[28]
iMSC and hSynovium-MSC	Not defined	Collagenase induced in mice	8 μL injection once per week for 21 days of iMSC-EVs (1.0 × 10^10^/mL) or MSC-EVs (1.0 × 10^10^/mL) or PBS	Healthy, OA	4 weeks	Histology (H), immunohistochemistry (IH), OARSI scores	The injection of iMSC-EVs and MSC-EVs both attenuated OA in the mouse OA model, but iMSC EVs had a superior therapeutic effect compared with MSC-EVs	[53]
hSynovium-MSC	Not defined	ACL rupture in rabbits and rats	For rabbits: 12 mg once per week of TA, T-NP, T-RNP, CD90@MV, T-CD90@NP For rats: 0.25 mg once per week of TA, T-NP, T-RNP, CD90@MV, T-CD90@NP	Sham, OA	24 weeks for rabbits and 2 weeks for rats	Histology (H), immunohistochemistry (IH), micro-CT, RNAseq	- CD90 EVs enhanced repair of damaged cartilage and effective anti-inflammatory ability - CD90 EVs promoted the regeneration of chondrocytes, reduced apoptosis via the FOXO pathway, and influenced type 2 macrophage polarization to regulate inflammation through IL-10	[47]
hSynovium-MSC	miR-26a-5p	DMM in rats	30 μL injection per week of GW inhibitor or EVs or EV-NC or EV-inhibitor (10^11^ particles/mL) or PBS	Sham, OA	4 weeks	Histology (H), ELISA, qPCR	miR-26a-5p MSC EVs inhibit apoptosis and inflammation and ameliorate cartilage damage of OA	[48]
hSynovium-MSC	miR-155-5p	DMM in mice	30 μL injection of MSC-EVs (10^11^ EVs particles/mL) or MSC-155-5p-EVs (10^11^ EVs particles/mL)	Healthy, OA	2 weeks	Histology (H), immunohistochemistry (IH), OARSI scores	miR-155-5p EVs prevent osteoarthritis via enhancing proliferation and migration, attenuating apoptosis, and modulating extracellular matrix secretion in chondrocytes	[49]
hSynovium-MSC	circRNA3503	DMM in rats	100 μL injection of PLEL@SMSC-EVs or PLEL@Wnt5a/b-dKO-EVs or PLEL@circRNA3503-OE-EVs or PLEL@dKO-OE-EVs or PLEL@Saline	Healthy, OA	24 weeks	Histology (H)	- circRNA3503-OE-EVs alleviate inflammation-induced apoptosis and the imbalance between ECM synthesis and ECM degradation - circRNA3503-OE-EVs promote chondrocyte renewal to alleviate the progressive loss of chondrocytes	[50]
hSynovium-MSC	Not defined	DMM in mice	10 μL injection twice weekly of PBS-EVs (10^11^ particles/mL) or PBS-LPS-pre EVs (10^11^ particles/mL) or PBS	Sham, PBS	6 weeks	Histology (H), immunohistochemistry (IH), OARSI scores	EVs derived from LPS-preconditioned MSC inhibit extracellular matrix degradation and prevent osteoarthritis	[51]
hSynovium-MSC	miR-140-5p	DMM in rats	100 μL injection of Synovium-MSC-EVs (10^11^ EVs particles/mL) or SMSC-140 EVs (10^11^ EVs particles/mL)	Healthy, OA	12 weeks	Histology (H), immunohistochemistry (IH), OARSI scores	miR-140-5p EVs enhance cartilage tissue regeneration and prevent osteoarthritis	[52]
hSynovium-MSC	miR-31	DMM in mice	5 μL injection every 3 days for 4 weeks of Synovium-MSC-EVs or EVs (miR-31 mimic) or PBS	Sham, OA	12 weeks	Histology (H), immunohistochemistry (IH), OARSI scores, ELISA	MSC EVs and EVs from miR-31-overexpressed MSC alleviated cartilage damage and inflammation in knee joints in vivo	[54]
hUC-MSC	LncRNA H19	Osteochondral defect in rats	100 μL injection once per week of EVs from UC-MSC transfected with siRNA H19 (si-EVs, 1 mg/mL) or EVs from UC-MSC with mechanical stimulation (S-EVs, 1 mg/mL) or PBS	PBS	4 and 8 weeks	Behavioral analysis, histology (H), MRI, ICRS scores	LncRNA H19 relieve pain levels during the early stages of cartilage repair via enhanced chondrocyte proliferation and matrix synthesis	[55]
hUC-MSC	miR-1208	DMM in mice	10 μL injection twice per week of MSC-EVs (10^11^ particles/mL) or antagomiR-NC (200 nmol/mL) or antagomiR-1208 (200 nmol/mL) or PBS	Sham, PBS	6 weeks	Histology (H), immunohistochemistry (IH), micro-CT, ELISA	MSC-EVs inhibited the secretion of proinflammatory factors and the degradation of cartilage ECM	[56]
hUC-MSC	circHIPK3	Collagenase induced in mice	Single injection of MSC-EVs or MSC-circHIPK3-EVs or circHIPK3 or PBS	Healthy, OA	Not reported	Histology (H), immunohistochemistry (IH), WB, qPCR	MSC-circHIPK3 EVs inhibited cartilage degradation	[61]

hAD-MSC: adipose tissue-derived MSC, hBM-MSC: bone marrow-derived MSC, hAFSC: amniotic fluid-derived MSC, hESC-MSC: embryonic stem cell-derived MSC, hIFP-MSC: infrapatellar fat pad-derived MSC, hSynovium-MSC: synovium-derived MSC, hUC-MSC: umbilical cord-derived MSC.

**Table 2:** Summary of MSC-EV-based clinical trials in OA therapy.

EV Source	Patients	Dose	Type of Trial	Delivery Method	Follow Up Period	Primary Outcome	Trial ID
Allogeneic UC MSCs	KL 2–3 knee OA, *n* = 10	3–5 × 10^11^ particles	Phase 1, safety and efficacy trial	Single IA injection	12 months	Adverse events, pain and disability reduction	NCT05060107
Allogeneic UC MSCs	KL 2–3 knee OA, *n* = 12, *n* = 4 per group	2 × 10^9^ particles/dose; 6 × 10^9^ particles/dose; 2 × 10^10^ particles/dose	Phase 1, open-label dose-escalation trial	Single IA injection	12 months	Adverse events, pain and disability reduction, percentage of responders at 52 weeks	NCT06431152
Autologous SF-MSCs	Bilateral degenerative meniscus, early OA, 3 groups of *n* = 10	EVs from 10^6^ SF MSCs; 10^6^ SF MSCs; control	Phase 2, randomized safety and efficacy trial	Single IA injection	12 months	Adverse events, pain reduction, cytokine measurements, knee motion and physical activity	NCT05261360
Allogeneic MSCs	KL 1–3 knee OA in both knees, *n* = 20	Not reported	Phase 1, safety and efficacy trial	IA injection, day 1 and day 90	1, 3, and 6 months	Adverse events, evaluation of pain, measurements of knee function	NCT06466850
Platelets: PEP (Purified EVs Product); 2 doses; with or without 1% sodium hyaluronate	KL 2–3 knee OA, *n* = 24	1 or 2 vials	Phase 1, randomized safety and exploratory efficacy dose-escalation trial	Single IA injection	90 days (primary safety); 12 months (long-term safety)	Primary and long-term safety, clinical improvements after 12 months	NCT06463132

## Data Availability

Not applicable.
